# CT whole lung radiomic nomogram: a potential biomarker for lung function evaluation and identification of COPD

**DOI:** 10.1186/s40779-024-00516-9

**Published:** 2024-02-20

**Authors:** Tao-Hu Zhou, Xiu-Xiu Zhou, Jiong Ni, Yan-Qing Ma, Fang-Yi Xu, Bing Fan, Yu Guan, Xin-Ang Jiang, Xiao-Qing Lin, Jie Li, Yi Xia, Xiang Wang, Yun Wang, Wen-Jun Huang, Wen-Ting Tu, Peng Dong, Zhao-Bin Li, Shi-Yuan Liu, Li Fan

**Affiliations:** 1 Department of Radiology, the Second Affiliated Hospital of Naval Medical University, Shanghai, 200003 China; 2 School of Medical Imaging, Shandong Second Medical University, Weifang, 261053 Shandong China; 3 Department of Radiology, School of Medicine, Tongji Hospital, Tongji University, Shanghai, 200065 China; 4 Department of Radiology, Zhejiang Province People’s Hospital, Affiliated People’s Hospital of Hangzhou Medical College, Hangzhou, 310014 China; 5 Department of Radiology, Sir Run Run Shaw Hospital, Zhejiang, 310018 China; 6 Jiangxi Provincial People’s Hospital, the First Affiliated Hospital of Nanchang Medical College, Nanchang, 330006 China; 7 College of Health Sciences and Engineering, University of Shanghai for Science and Technology, Shanghai, 200093 China; 8 Department of Radiology, the Second People’s Hospital of Deyang, Deyang, 618000 Sichuan China; 9 Department of Radiation Oncology, Shanghai Jiao Tong University Affiliated Sixth People’s Hospital, Shanghai, 200233 China

**Keywords:** Chronic obstructive pulmonary disease (COPD), Computed tomography (CT), Radiomic

## Abstract

**Background:**

Computed tomography (CT) plays a great role in characterizing and quantifying changes in lung structure and function of chronic obstructive pulmonary disease (COPD). This study aimed to explore the performance of CT-based whole lung radiomic in discriminating COPD patients and non-COPD patients.

**Methods:**

This retrospective study was performed on 2785 patients who underwent pulmonary function examination in 5 hospitals and were divided into non-COPD group and COPD group. The radiomic features of the whole lung volume were extracted. Least absolute shrinkage and selection operator (LASSO) logistic regression was applied for feature selection and radiomic signature construction. A radiomic nomogram was established by combining the radiomic score and clinical factors. Receiver operating characteristic (ROC) curve analysis and decision curve analysis (DCA) were used to evaluate the predictive performance of the radiomic nomogram in the training, internal validation, and independent external validation cohorts.

**Results:**

Eighteen radiomic features were collected from the whole lung volume to construct a radiomic model. The area under the curve (AUC) of the radiomic model in the training, internal, and independent external validation cohorts were 0.888 [95% confidence interval (CI) 0.869–0.906], 0.874 (95%CI 0.844–0.904) and 0.846 (95%CI 0.822–0.870), respectively. All were higher than the clinical model (AUC were 0.732, 0.714, and 0.777, respectively, *P* < 0.001). DCA demonstrated that the nomogram constructed by combining radiomic score, age, sex, height, and smoking status was superior to the clinical factor model.

**Conclusions:**

The intuitive nomogram constructed by CT-based whole-lung radiomic has shown good performance and high accuracy in identifying COPD in this multicenter study.

**Supplementary Information:**

The online version contains supplementary material available at 10.1186/s40779-024-00516-9.

## Background

Chronic obstructive pulmonary disease (COPD) is a chronic inflammatory disorder with high heterogeneity and characterized by continuous airflow limitations. The current gold standard for diagnosing and evaluating COPD is the pulmonary function test (PFT) [1], which yields the ratio of forced expiratory volume in 1 s to forced vital capacity (FEV1/FVC) and the percentage of predicted FEV1 (FEV1% predicted). In China, the incidence of COPD in people ≥ 40 years old is 13.7%, however, the awareness rate of COPD is very low, less than 1% [2]. Many people have been underdiagnosed because the PFT is not widely used for screening in China. Based on the survey of PFT performance in people aged 40 years and above in China, the PFT rate in Chinese residents aged ≥ 40 years was 6.7% (95%CI 5.2–8.2%) in 2019–2020, the overall PFT rate was still at a low level [3]. In contrast, the popularity of chest computed tomography (CT) is high, especially with large-scale lung cancer screening. Meanwhile, the 2023 Global Initiative for Chronic Obstructive Lung Disease report emphasized the importance of CT in evaluating patients with stable COPD, highlighting the role of imaging [4]. This evidence-based suggestion was made due to the limitations of PFT, which, despite being the gold standard, cannot be used for focal evaluation and does not show the lungs. Additionally, due to the high heterogeneity of COPD, focal and visual evaluations are considered to play an important role in guiding clinical decisions. As the most common and powerful imaging technique, CT has great potential in COPD with the rapid development of CT-based artificial intelligence (AI).

Furthermore, CT has a high anatomic resolution and can be used to evaluate the changes in lung parenchyma, small airway, and pulmonary blood vessels that occur with lung function decline and aging. However, it is not commonly used to simultaneously assess COPD abnormalities. In addition, the subjective evaluation of the lung parenchyma and small airway lesions was influenced by the experience of the radiologist. Especially as the number of patients with COPD continues to increase, visual assessment of lung lesions by radiologists is becoming more expensive and laborious. Therefore, identifying a method for quantitatively evaluating the whole lung is critical to obtaining a comprehensive evaluation of COPD.

Radiomic is a relatively novel approach that can rapidly collect quantitative high-throughput features from medical images (e.g., CT), such as complex patterns that are not easily recognized or quantified by the naked eye [5], showing great potential in clinical decision-making. At present, radiomic research on COPD is rapidly expanding, and several studies have indicated that such a method may have particular advantages in patients with COPD [6, 7]. The radiomic features are extracted from segmented lesions. However, radiologists have found that manually segmenting diffuse and heterogeneous lung lesions, such as emphysema, interstitial lung disease, and coronavirus disease 2019 [8, 9], are difficult and time-consuming due to unclear boundaries or low contrast on CT imaging. Additionally, different radiologists may segment the lesions differently when evaluating diffuse lung diseases [10]. Therefore, it is very important to develop a method to automatically segment diffuse lesions. It has been reported that the application of an AI-based system to detect diseases can reduce the workload of radiologists and maintain the accuracy of diagnoses [11]. Because COPD is a diffuse chronic lung disease, automatic segmentation of the whole lung region would help to comprehensively quantify lung abnormalities and aid clinical treatment decision-making. A recent editorial suggests that automated detection of COPD based on chest CT findings using radiomic or deep learning techniques has great potential to reduce the current underdiagnosis of COPD, particularly in high-risk cohorts [12]. The purpose of this study was to explore the performance of CT-based automatic segmentation of whole-lung radiomic in differentiating COPD from non-COPD and to assess the value of CT-based radiomic in lung function evaluation.

## Materials and methods

### Patients

**Fig. 1:**
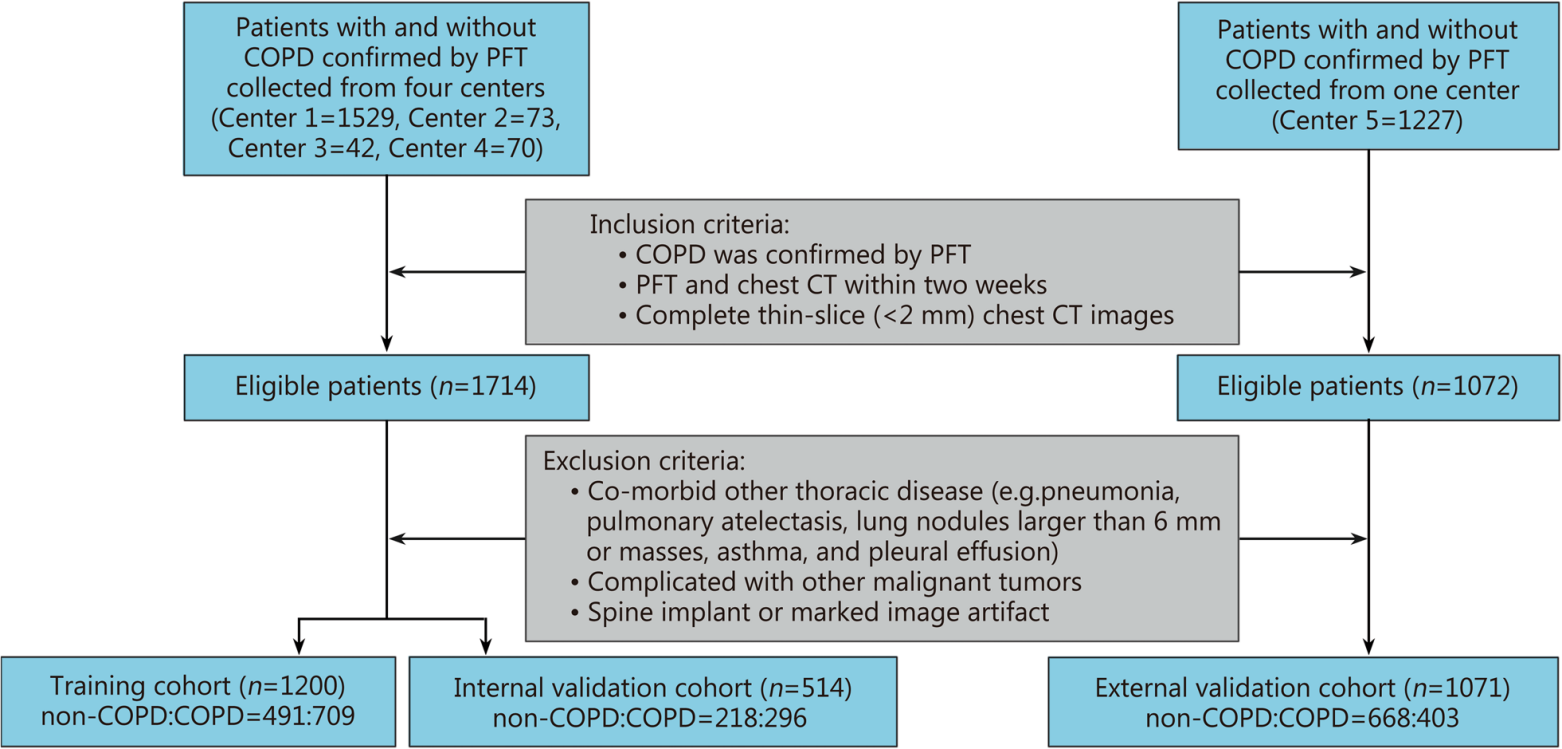
Diagram showing the patient inclusion and exclusion process. Center 1: Tongji Hospital, School of Medicine, Tongji University; Center 2: Zhejiang Province People’s Hospital; Center 3: Sir Run Run Shaw Hospital; Center 4: the First Affiliated Hospital of Nanchang Medical College; Center 5: the Second Affiliated Hospital of Naval Medical University. COPD chronic obstructive pulmonary disease, PFT pulmonary function disease, CT computed tomography

A total of 2941 patients who were admitted and underwent PFT at 5 centers, including the Second Affiliated Hospital of Naval Medical University, Tongji Hospital, School of Medicine, Tongji University, Zhejiang Province People’s Hospital, Sir Run Run Shaw Hospital and the First Affiliated Hospital of Nanchang Medical College, were retrospectively recruited from February 2013 to December 2022 in the CSD-COPD cohort. The inclusion criteria were as follows: 1) chest CT and PFT both performed in the same hospital; 2) less than two weeks between PFT and chest CT; and 3) complete thin-slice (< 2 mm) chest CT images. The exclusion criteria were as follows: 1) other comorbid thoracic diseases (e.g., pneumonia, pulmonary atelectasis, lung nodules larger than 6 mm or masses, asthma, and pleural effusion); 2) malignant tumors; and 3) spine implants or substantial image artifacts. Finally, 2785 patients were included in this study. Among them, 1714 patients from the Tongji Hospital, School of Medicine, Tongji University, Zhejiang Province People’s Hospital, Sir Run Run Shaw Hospital, and the First Affiliated Hospital of Nanchang Medical College were randomly assigned to the training cohort (*n* = 1200) and the internal validation cohort (*n* = 514) in a ratio of 7:3. Patients from the Second Affiliated Hospital of Naval Medical University were assigned to an independent external validation cohort (*n* = 1071). Figure 1 shows the workflow for patient inclusion and exclusion. The basic clinical information of the patients, including age, sex, weight, height, body mass index, and smoking status, was collected through the electronic medical records system.

Pulmonary function parameters (FEV1, FVC) were measured with PFT apparatus (CHEST Multifunction Spirometer HI-801, Japan; Ganshorn Medizin Electronic GmbH; Carefusion GmbH, Hoechberg, Germany; Masterscreen PFT Pro, Carefusion, Netherlands), as well as CT acquisition parameters in Additional file 1: Table S1. The diagnostic criteria for PFT in COPD are as follows: FEV1/FVC < 0.7 with an increase of FEV1 < 200 ml after the use of a bronchodilator. In contrast, this study included patients with FEV1/FVC ≥ 0.7 and the FEV1% predicted ≥ 80% after bronchodilation as the non-COPD group. Participants in the training, internal validation, and independent external validation cohorts were divided into COPD and non-COPD groups according to these criteria.

This study was approved by the institutional review boards at 5 centers, and informed consent was waived due to the retrospective nature of this study (ChiCTR2300069929).

### Whole-lung CT image segmentation and CT image preprocessing

**Fig. 2:**
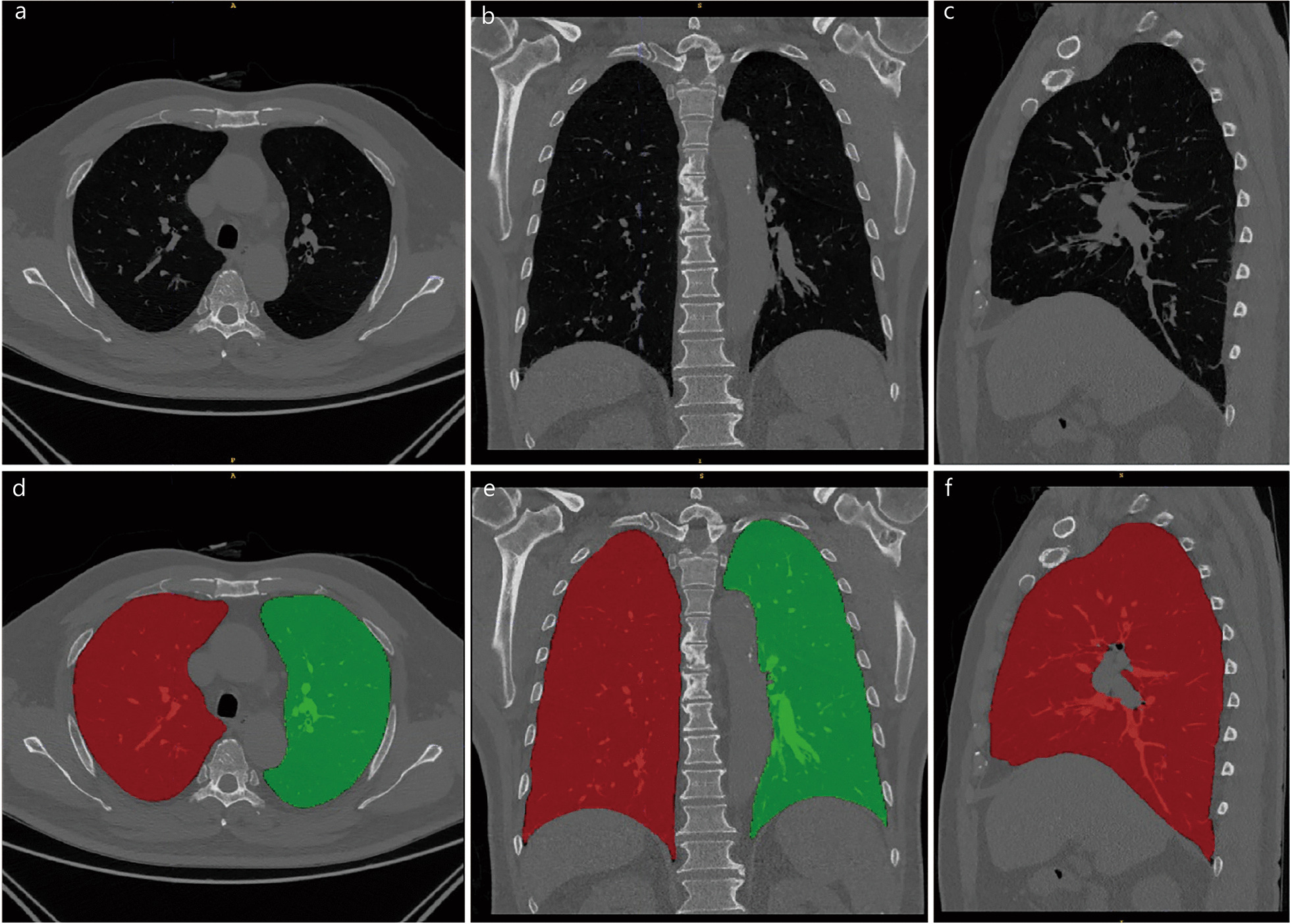
Original chest HRCT images (**a–c**) and segmentation results (**d–f**) of typical lung regions in transverse, coronal, and sagittal planes based on the original chest HRCT images, respectively. The red mask is the right lung parenchyma, and the green one is the left lung parenchyma. HRCT high-resolution computed tomography

Using a deep-learning model of open access U-net (R231) (https://github.com/JoHof/lungmask) for the automatic segmentations, which has been trained using different large-scale datasets covering a wide range of visual variability, the reliability of this method has been proved [13]. First, the right and left lungs were automatically segmented. Then, we merged the right and left lungs into a combined region of interest (ROI) (Fig. 2).

Since manual segmentation is often regarded as the ground truth, we assessed the consistency between manual and fully automatic segmentation in 20 randomly selected patients across the cohort. CT images of 20 patients were exported to ITK-SNAP software (version 3.8.0, www.itksnap.org) for manual segmentation. The consistency between manual and fully automatic segmentation was assessed using the Dice index, an objective measure that quantifies the spatial overlap between two contours. The remaining cases were then automatically segmented.

Before extracting the radiomic features, the images were preprocessed, which consisted of three steps. First, we used linear interpolation to resample the images to 1 mm × 1 mm × 1 mm. Second, we used gray-level discretization to convert continuous images into discrete integer values. Finally, log and wavelet image filters were used to eliminate the mixed noise in the process of image digitization and obtain low-frequency or high-frequency features.

### Radiomic feature extraction and selection

A total of 1218 lung radiomic features were extracted from each volume of interest using the open-source package PyRadiomics (version 3.0.1, https://pyradiomics.readthedocs.io/en/latest/), including first-order, gray level co-occurrence matrix, gray level run length matrix, gray level size zone matrix, gray level dependence matrix, and shape features. The radiomic features extracted by this software are in accordance with the image biomarker standardization initiative. The Z score method was used to normalize the features and eliminate the difference in numerical scale.

The following three steps were used to select the best radiomic features. First, redundant features whose correlation coefficient with other features is greater than 0.90 were removed. Second, the maximal redundancy-minimal relevance algorithm was used to eliminate the redundant and irrelevant features. Minimal redundancy maximal relevance has been proven to be an effective and reliable feature selection method for radiomic, which can consider both the importance of features and the correlation between features to find the optimal feature subset [14, 15]. Finally, the least absolute shrinkage and selection operator (LASSO) regression algorithm and penalty parameter adjustment were used for tenfold cross-validation. The optimal feature dataset with the smallest cross-validation binomial deviation was selected, and the non-zero coefficients were defined as the weight of the selected feature, representing the correlation between the feature and COPD. LASSO is a widely used embedded method for radiomic feature selection in high-dimensional data [16]. Finally, the Radscore of each patient was calculated by a linear combination of the selected feature and coefficient vectors, and the radiomic model was constructed.

### Model construction, radiomic nomogram, and performance evaluation

Three models were constructed, including the clinical model, radiomic model, and combined model. Univariate logistic regression analysis was used to obtain statistically significant risk variables, and then multivariate analysis was performed to establish clinical and combined models. A radiomic nomogram was generated to visualize the combined model, graphically evaluate variable importance, and calculate prediction accuracy. The DeLong test was used to compare the area under the curves (AUCs) of the clinical model, radiomic model, and combined model. The calibration curves (Hosmer–Lemeshow test) were performed to evaluate the calibration of the nomogram. Decision curve analysis (DCA) was applied to evaluate the clinical practicability of the nomogram.

### Statistical analysis

IBM SPSS Statistics (version 26.0; IBM Corp., New York, USA) and R software (version 4.2.2; http://www.Rproject.org) were employed for statistical analysis. Measurement variables are expressed as the mean ± standard deviation. Normally distributed continuous variables were compared using the Student’s unpaired *t*-test and non-normally distributed data were compared using the Mann–Whitney U test. Categorical variables were compared by the chi-square test between groups. Independent predictors were identified from the clinical variables by multivariate logistic regression. *P* < 0.05 indicated statistical significance. LASSO regression was conducted using the “glmnet” package. Additionally, the “rms” package was employed for drawing calibration plots and conducting multivariate logistic regression. The package of receiver operating characteristic (ROC) was utilized for drawing the ROC curves of the radiomic signatures, while the “rmda” package was utilized for DCA.

## Results

### Clinical characteristics

In total, 2785 patients (male 1715, female 1070; non-COPD group 1377, COPD group 1408) with an average age of (65.4 ± 11.4) years old were included. Table 1 displays the basic demographics of all patients studied. In the training and internal validation cohorts, the distribution of the patients in the 4 independent centers was as follows: 1529 patients from Tongji Hospital, School of Medicine, Tongji University, 73 patients from Zhejiang Province People’s Hospital, 42 patients from Sir Run Run Shaw Hospital and 70 patients from the First Affiliated Hospital of Nanchang Medical College. The training cohort included 491 non-COPD patients and 709 COPD patients, and the internal validation cohort included 218 non-COPD patients and 296 COPD patients. A total of 1071 patients from the Second Affiliated Hospital of Naval Medical University were assigned to the independent external validation cohort, consisting of 668 patients without COPD and 403 patients with COPD. Significant differences were observed in age, sex, height, weight, body mass index, and smoking status between the non-COPD and COPD groups (*P* < 0.05) in the training, internal and independent external cohorts. However, in the internal validation cohort, the difference between current and former smokers was not significant **(**Table 1**)**.

**Table 1:** Baseline characteristics of the study population

Characteristic	Training cohort (*n* = 1200)			Internal validation cohort (*n* = 514)			External validation cohort (*n* = 1071)		
	Non-COPD (*n* = 491)	COPD (*n* = 709)	*P*-value	Non-COPD (*n* = 218)	COPD (*n* = 296)	*P*-value	Non-COPD (*n* = 668)	COPD (*n* = 403)	*P*-value
Age (years, mean ± SD)	63.9 ± 0.5	70.0 ± 0.4	< 0.001	64.1 ± 0.7	69.2 ± 0.6	< 0.001	58.9 ± 0.5	67.5 ± 0.5	< 0.001
Sex [*n* (%)]			< 0.001			< 0.001			< 0.001
Male	232 (47.3)	509 (71.8)		104 (47.7)	214 (72.3)		326 (48.8)	330 (81.9)	
Female	259 (52.7)	200 (28.2)		114 (52.3)	82 (27.7)		342 (51.2)	73 (18.1)	
Height (cm, mean ± SD)	160.9 ± 0.5	163.9 ± 0.3	< 0.001	160.9 ± 0.6	163.9 ± 0.5	< 0.001	161.2 ± 0.3	163.3 ± 0.4	< 0.001
Weight (kg, mean ± SD)	63.7 ± 0.5	65.4 ± 0.4	0.003	63.5 ± 0.8	64.6 ± 0.7	0.270	62.4 ± 0.4	63.6 ± 0.6	0.132
BMI (kg/m^2^, mean ± SD)	25.3 ± 0.6	24.3 ± 0.2	0.462	24.5 ± 0.3	24.0 ± 0.2	0.153	24.0 ± 0.1	23.8 ± 0.2	0.251
Smoking status [*n* (%)]			< 0.001			< 0.001			< 0.001
Non-smoker	397 (80.9)	416 (58.7)		176 (80.7)	183 (61.8)		527 (78.9)	207 (51.4)	
Former smoker	13 (2.6)^a^	90 (12.7)^a^		10 (4.6)^a^	35 (11.8)^a^		44 (6.6)^a^	88 (21.8)^a^	
Current smoker	81 (16.5)^a,b^	203 (28.6)^a,b^		32 (14.7)^a^	78 (26.4)^a^		97 (14.5)^a,b^	108 (26.8)^a,b^	

^*a*^*P* < 0.05 vs. non-smoker, ^*b*^*P* < 0.05 vs. former smoker. *COPD* chronic obstructive pulmonary disease, *BMI* body mass index, *SD* standard deviation

### Consistency assessment between manual and fully automatic segmentation

The segmentations were assessed using the Dice index, an objective measure that quantifies the spatial overlap between two contours. The mean Dice coefficient between manual and automatic segmentation was (0.97 ± 0.06) (Additional file 1: Fig. S1).

### Feature screening and radiomic signatures establishment

**Fig. 3:**
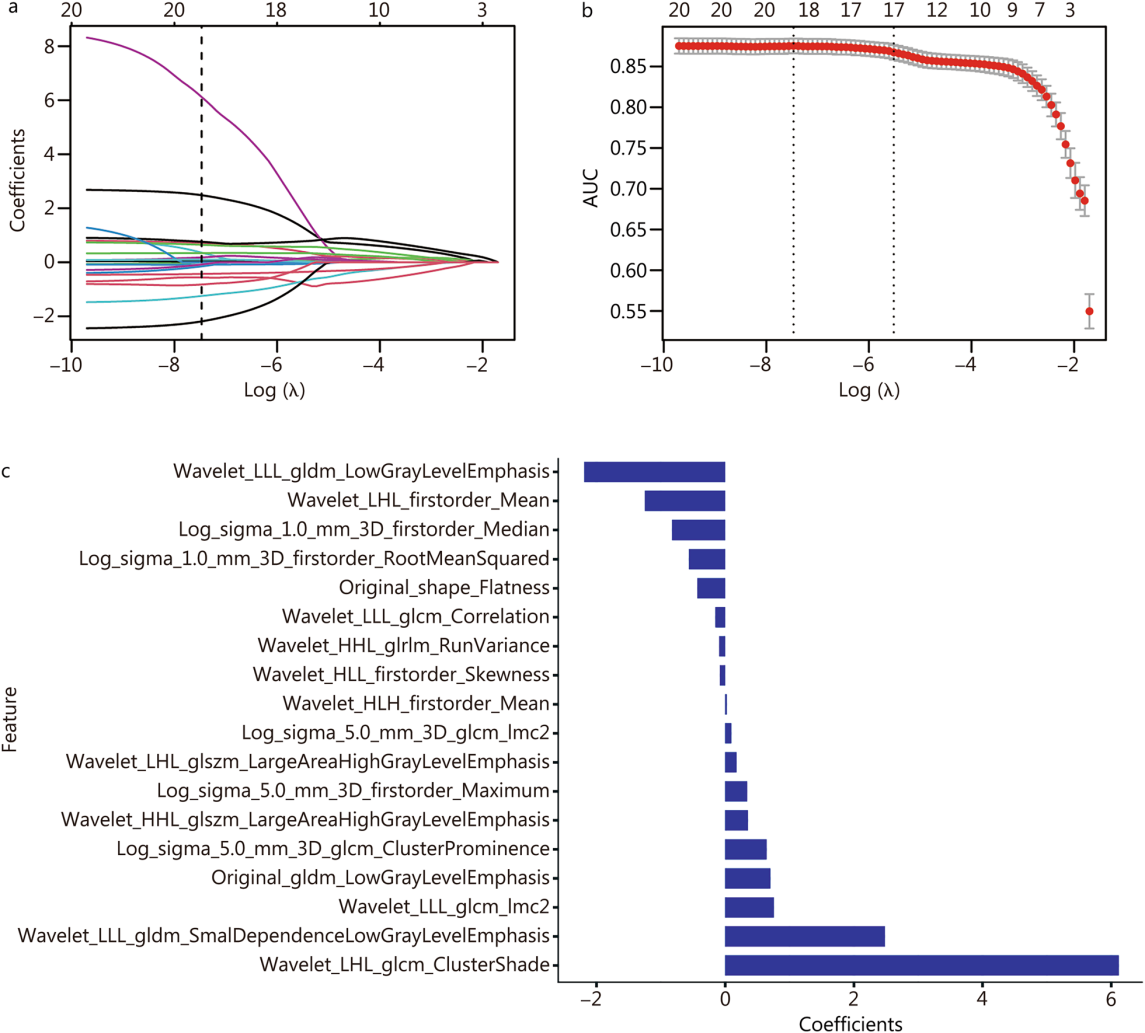
LASSO coefficients of radiomic features. **a** The LASSO coefficient profiles of the 283 radiomics features. A vertical line was generated at the log (λ) value by using tenfold cross-validation, where the optimal λ value resulted in 18 radiomics features. The optimal λ value of 0.00057 was selected. The X-axis on the top indicates the number of nonzero coefficient features in the model. **b** The black vertical line was drawn at the value selected using tenfold cross-validation in (**a**). The X-axis on the top indicates the number of nonzero coefficient features in the model. **c** Histogram of the Radscore: the Y-axis indicates the selected 18 radiomic features, and the X-axis represents the coefficient of the radiomic features. LASSO least absolute shrinkage and selection operator

A total of 1218 radiomic features were normalized with the Z score method. After Pearson’s correlation analysis, 935 radiomic features (absolute value of Pearson correlation coefficients > 0.9) were eliminated. Therefore, a total of 283 features were retained. Finally, 18 radiomic features with non-zero coefficients were selected by LASSO regression (Fig. 3a–c). By linearly combining those features after weighting by their corresponding coefficients, we constructed the radiomic signature. The Radscore calculation formula is provided in the Additional file 1.

### Performance comparison of radiomic model, clinical model, and combined model

The boxed scatter plots for the Radscore are shown in the Additional file 1: Fig. S2. As revealed by the Wilcoxon test, the Radscore exhibited significant differences between the COPD group and the non-COPD group (*P* < 0.001). In addition, according to univariate and multivariate regression analysis, the Radscore was independently associated with COPD. Moreover, age, sex, height, and smoking status were identified as independent predictors of COPD by multivariate regression and included in the construction of the clinical model (Table 2). Last, the Radscore was integrated with these independent predictive factors to construct the combined model. The combined model calculation formula is described in Additional file 1.

**Table 2:** Univariable and multivariable logistic regression analysis

Variable	Univariable analysis		Multivariable analysis (clinical model parameters)		Multivariable analysis (combined model parameters)	
	*OR* (95%CI)	*P*-value	*OR* (95%CI)	*P*-value	*OR* (95%CI)	*P*-value
Age	1.059 (1.046–1.072)	< 0.001	1.074 (1.059–1.089)	< 0.001	1.025 (1.007–1.043)	0.007
Sex	0.352 (0.276–0.447)	< 0.001	0.663 (0.459–0.957)	0.028	1.839 (1.151–2.937)	0.011
Height	1.034 (1.021–1.047)	< 0.001	1.029 (1.010–1.050)	0.003	1.022 (0.999–1.045)	0.061
Weight	1.012 (1.002–1.022)	0.014	**–**	**–**	**–**	**–**
BMI	0.986 (0.967–1.006)	0.166	**–**	**–**	**–**	**–**
Smoking	0.599 (0.516–0.695)	< 0.001	0.660 (0.554–0.785)	< 0.001	0.662 (0.531–0.826)	< 0.001
Radscore	2.862 (2.511–3.263)	< 0.001	–	–	2.789 (2.410–3.227)	< 0.001

*BMI* body mass index, *OR* odds ratios, *CI* confidence interval

**Fig. 4:**
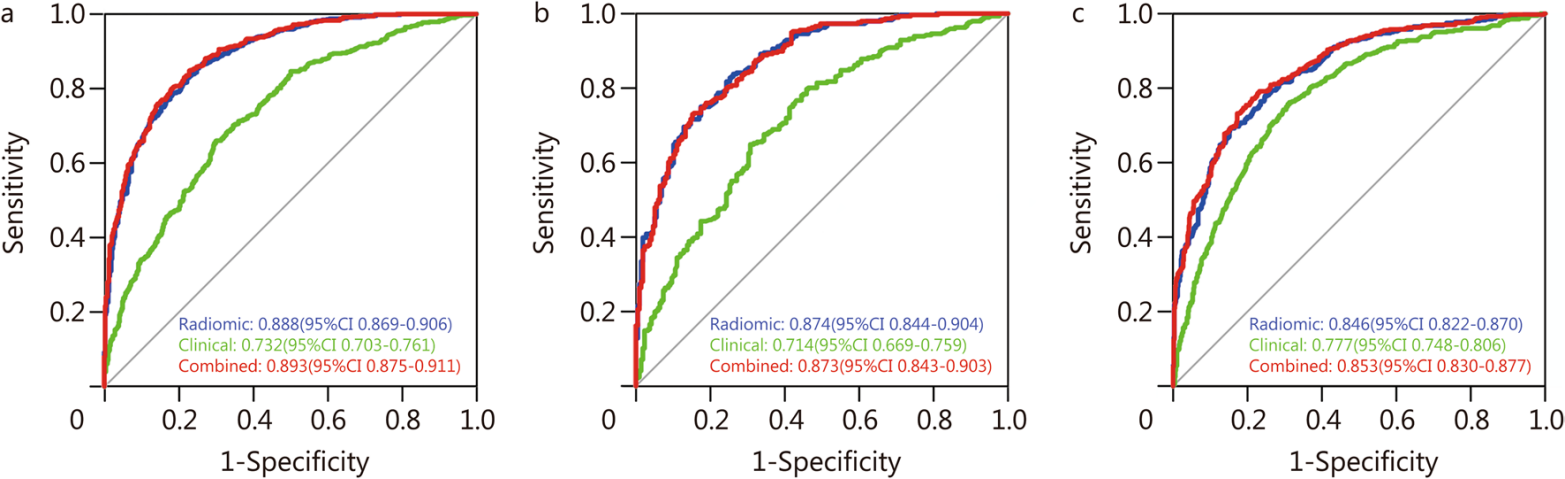
ROC curves of the radiomic model, clinical model, and combined model in predicting COPD in the training cohort (**a**), internal validation cohort (**b**), and external validation cohort (**c**). ROC receiver operating characteristic, COPD chronic obstructive pulmonary disease

Figure 4 and Table 3 showed the performances of the radiomic, clinical, and combined models. The constructed radiomic model contains 18 screened features and had a good degree of differentiation, with AUCs of 0.888 (95%CI 0.869–0.906), 0.874 (95%CI 0.844–0.904) and 0.846 (95%CI 0.822–0.870) in the training, internal and external validation cohorts, respectively. According to the DeLong test, there was a significant difference in the AUCs between the combined model and the clinical model (*P* < 0.001 in the three cohorts). The DeLong test also showed that the AUC of the combined model and the radiomic model in the training cohort was significantly different [AUC = 0.893 (95%CI 0.875–0.911) vs. AUC = 0.888 (95%CI 0.869–0.906); *P* = 0.02] and in the external validation cohort [AUC = 0.853 (95%CI 0.830–0.877) vs. AUC = 0.846 (95%CI 0.822–0.870); *P* = 0.04], but there was no significant difference in the internal validation cohort [AUC = 0.873 (95%CI 0.843–0.903) vs. AUC = 0.874 (95%CI 0.844–0.904); *P* = 0.71].

**Table 3:** Comparison of diagnostic performance of the radiomic model, clinical model, and combined model in the training and internal and external validation cohorts

Model	AUC (95%CI)	Accuracy (95%CI) (%)	Sensitivity (%)	Specificity (%)	PPV (%)	NPV (%)
Radiomic model						
Training cohort	0.888 (0.869 – 0.906)	81.3 (78.9 – 83.4)	85.5	75.2	83.2	78.2
Internal validation cohort	0.874 (0.844 – 0.904)	78.8 (75.0 – 82.3)	81.1	75.7	81.9	74.7
External validation cohort	0.846 (0.822 – 0.870)	76.9 (74.3 – 79.4)	54.3	90.6	77.7	76.7
Clinical model						
Training cohort	0.732 (0.703 – 0.761)	67.6 (64.9 – 70.2)	65.8	70.1	76.1	58.7
Internal validation cohort	0.714 (0.669 – 0.759)	65.4 (61.1 – 69.5)	62.5	69.3	73.4	57.6
External validation cohort	0.777 (0.746 – 0.806)	72.6 (69.9 – 75.3)	62.7	78.5	63.8	77.7
Combined model						
Training cohort	0.893 (0.875 – 0.911)	81.7 (79.4 – 83.8)	84.7	77.2	84.3	77.8
Internal validation cohort	0.873 (0.843 – 0.903)	78.0 (74.2 – 81.5)	81.8	73.1	79.4	76.2
External validation cohort	0.853 (0.830 – 0.877)	78.3 (75.7 – 80.7)	88.5	61.5	79.2	76.3

*AUC* area under the curve, *CI* confidence interval, *PPV* positive predictive value, *NPV* negative predictive value

### Development and performance of the nomogram

**Fig. 5:**
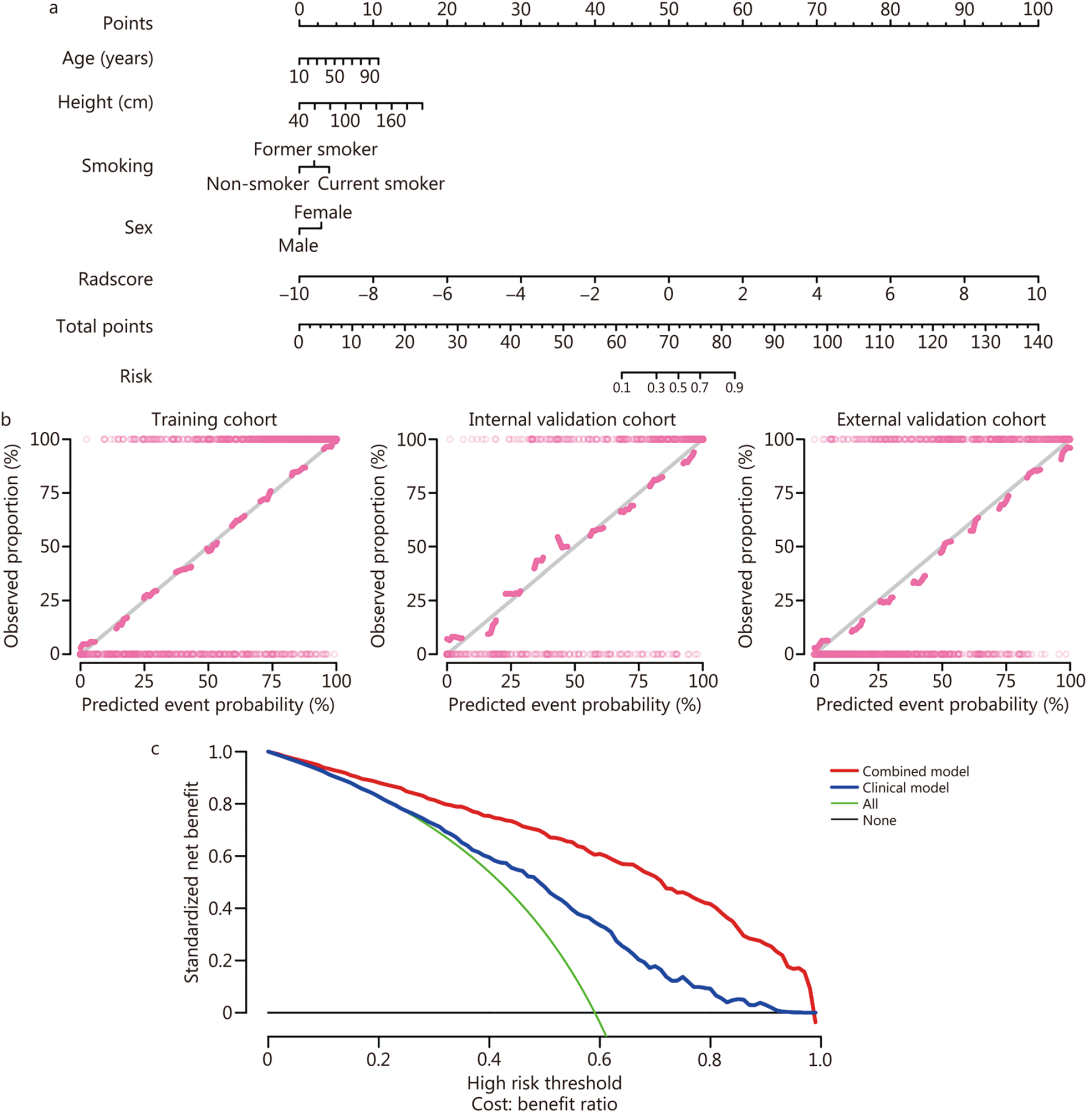
Development and performance of radiomic nomogram. **a** Radiomic nomogram developed to predict COPD. **b** Calibration curve between the predicted and actual incidences of COPD. **c** Decision curve analysis compares the net benefits of four scenarios in predicting the risk of COPD: Combined model (red line), Clinical model (blue line), All (green line, refers to the assumption that all patients have COPD) and None (horizontal solid black line, represents the assumption that no patient has COPD). COPD chronic obstructive pulmonary disease

**Fig. 6:**
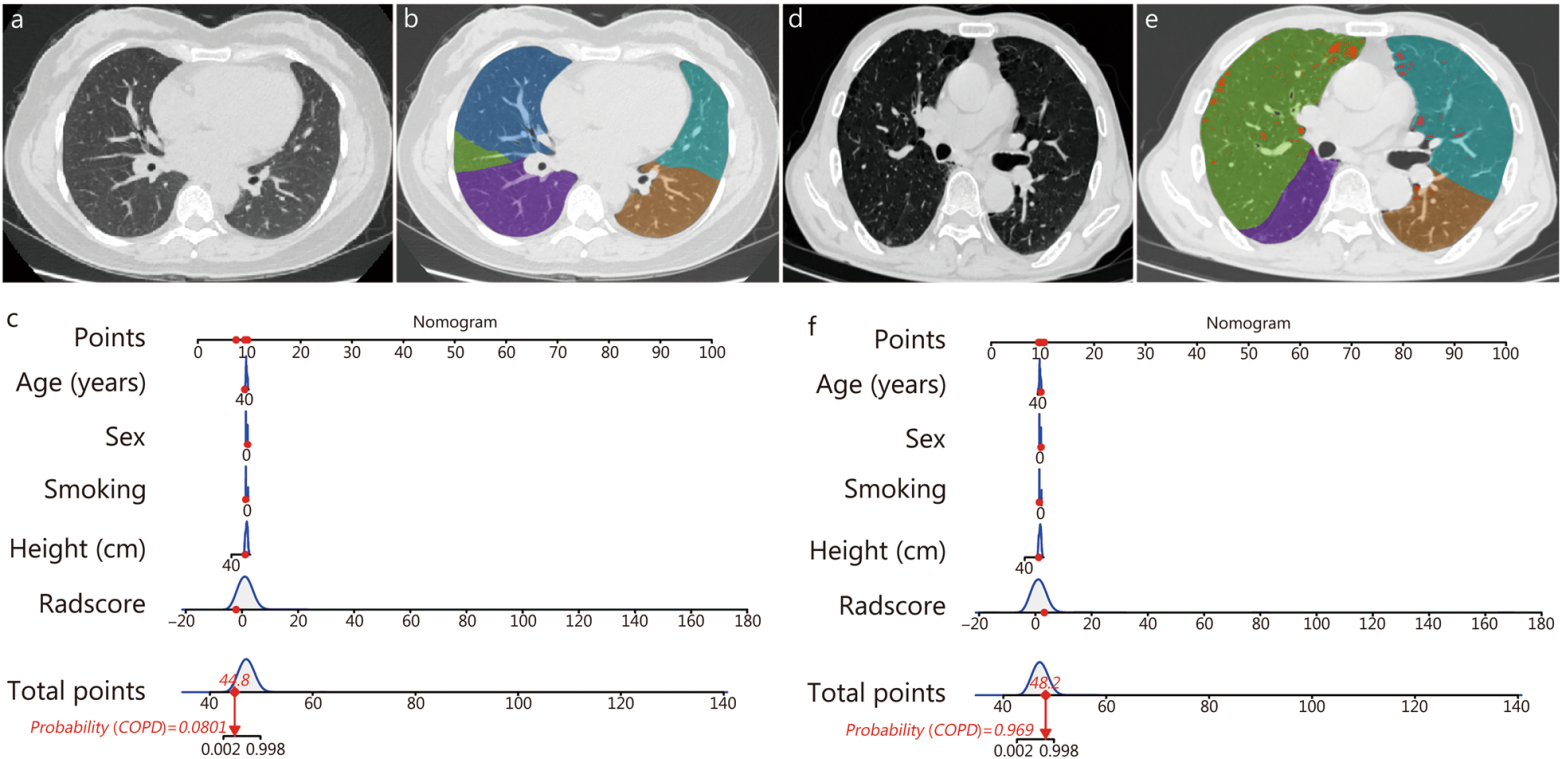
The risk scores of COPD in two patients were calculated by using the nomogram. **a** Thin-slice chest CT images of non-COPD in a 45-year-old woman with height 152 cm, non-smoker, Radscore -2.08. **b** Lung density analysis diagram showed no emphysema area in both lungs. **c** The nomogram shows that the total score was 44.8 points, corresponding to the probability of developing COPD is approximately 8.0%. Lung function examination showed that FEV1/FVC = 0.8. **d** Thin-slice chest CT image of COPD in an 82-year-old female subject. She is 152 cm tall, non-smoker, and has a Radscore of 3.17. **e** Lung density analysis diagram showed that both lungs are mostly scattered in the emphysema area (red). **f** The total score of the nomogram was 48.2, corresponding to the probability of developing COPD of approximately 96.9%. Pulmonary function examination showed that FEV1/FVC = 0.6. COPD chronic obstructive pulmonary disease, CT computed tomography, FEV1/FVC ratio of forced expiratory volume in 1 s to forced vital capacity

The visualization of the nomogram and the combination of radiomic and common clinical factors are helpful for doctors to conduct health education consultations for patients. The combined model was converted into a nomogram, and the total score obtained from the nomogram was used to predict the risk of COPD (Fig. 5a). The Hosmer–Lemeshow test showed that the calibration curves of the combined model for predicting COPD in the training, internal and external validation cohorts matched the actual data very well (*P* = 0.972, 0.149 and 0.06, respectively) (Fig. 5b). According to DCA (Fig. 5c), the combined model showed a greater benefit than the clinical model in predicting COPD risk in the training cohort when the probability threshold in the clinical decision of the patient or physician was greater than 0.1. The nomogram showed the highest clinical net benefit across all threshold probability ranges in the training cohort, suggesting that the nomogram is a reliable tool for clinically predicting COPD. An example of the nomogram in use is shown in Fig. 6. Similar to the points scoring system, we assigned points for each predictor of COPD and then equated these predictors with the risk of COPD. We can read the top score scale upward from the predictors to determine the points score associated with patient age, height, smoking status, sex, and the Radscore. Once a score has been assigned to each predictor, an overall score is calculated. Then, the total score is converted to the probability of COPD by reading the associated probability of COPD from the total point scale.

## Discussion

COPD is a heterogeneous disease that causes a series of abnormalities, including small airway remodeling, lung vessel remodeling, and the formation of emphysema. The ability to comprehensively evaluate the disease is very important. Although PFT is the current clinical gold standard, CT plays an important role in the management of COPD due to its advantages of focal, accurate, and visual evaluations. In our large multicenter cohort of participants, we innovatively proposed the construction of a CT-based whole lung radiomic nomogram to identify COPD. The AUCs of the model were 0.893, 0.873, and 0.853 in the training, the internal validation, and the independent external validation cohorts, respectively. The subsequently constructed nomogram is intuitive, which can improve the value of CT in evaluating lung function and help to detect more underdiagnosis of COPD in clinical routine work.

The incidence and disease burden of COPD is high in China, the overall pulmonary function detection rate is still at a low level, and many people have been underdiagnosed. In contrast, the popularity of chest CT is very high, especially with the large-scale chest CT screening for lung cancer. Moreover, more and more community health service centers will be equipped with CT. Therefore, the most important clinical scenario is for the large-scale lung cancer screening population that usually does not perform PFT, and many underdiagnosed COPD can be found through our model prediction, which can help enhance the detection and early intervention of COPD, reduce the socioeconomic burden and improve the patient’s life quality. A recent study revealed that CT-based radiomic features extracted only from inspiratory CT scans outperformed existing advanced methods in detecting COPD on both standard- and low-dose CT scans. The model was constructed with the standard-dose CT radiomic feature [17].

Radiomic has great potential in obtaining useful medical information and enhancing the accuracy of clinical differential diagnosis. A previous study has identified the value of lung radiomic features based on CT imaging and clinical manifestations in the assessment of COPD [7]. CT finding of COPD patterns might be obscure and diffuse, making it difficult to accurately delineate abnormal areas. Li et al. [18] randomly selected 42 non-overlapping ROIs from 11 axial CT sections of every patient to extract radiomic features, with an AUC of 0.97. However, the approach they used could not comprehensively evaluate the disease in the whole lung. In contrast, the automatic segmentation of the whole lung into the whole ROI allows a comprehensive evaluation of the lung, with an AUC of 0.893 in this study. Automatic segmentation can improve efficiency and reduce inter- and intra-observer differences. However, the AUC of COPD identified in our study was lower than that of Li et al. [18], which may be related to the fact that they applied machine learning technologies for further radiomic feature selection based on LASSO regression. Nam et al. [19] trained and validated a deep learning method to predict the prognosis of COPD patients based on chest radiography, with an AUC of 0.76. Notably, a significant proportion of patients with COPD had normal chest X-rays based on subjective evaluations. Compared to chest X-ray images, chest CT scans are more sensitive to changes in COPD. Therefore, chest CT was used to evaluate COPD in this study. The greatest characteristic of radiomic in this study was that the whole lung was combined into one ROI to extract the radiomic features. Because COPD is a diffuse and heterogeneous disease involving the pulmonary parenchyma, small airway, and lung blood vessels, focal ROIs cannot fully represent the pathological changes induced by the disease. Moreover, we used the deep convolutional neural network extension based on the U-net architecture for lung segmentation [13, 20].

According to our results, 283 potential radiomic features were selected on CT images, of which the LASSO regression model ultimately identified 18 predictors for constructing radiomic signatures. The radiomic features we screened were divided into four types (first-order, morphologic features, texture features, and wavelet features), which were significantly different between the non-COPD and COPD groups. These features essentially reflect information from the distribution of pixel intensity and texture morphology that radiologists cannot detect manually [21]. Morphologic features describe the size, volume, and shape of the volume of interest, while first-order features mainly reflect the internal texture of the lesions. Textural features, including the gray level co-occurrence matrix and gray level dependence matrix, describe the spatial relationship between each pixel and its neighbors. Wavelet features mainly reflect the time-frequency domain within the lesion [22]. Among the selected radiomic features, Wavelet LLL gldm LowGrayLevelEmphasis and wavelet LHL glcm ClusterShade have the highest significance and robustness in identifying COPD. They represent the intensity and textural features of lesions in high-intensity CT voxels. To some extent, radiomic is a quantitative method. Conventional quantitative CT evaluation has been applied in COPD diagnosis, severity evaluation, prognosis, and many other aspects. Cho et al. [23] reported the performance of an integrated model of quantitative features, such as emphysema, airway remodeling, pulmonary vascular diseases, and air trapping, extracted by fully automated in-house software (AVIEW) with a radiomic approach as a predictor of survival in COPD patients, and they found that their integrated model outperformed a model constructed using only a single quantitative parameter. In our research, we established a whole-lung radiomic signature describing airways, blood vessels, and emphysema, similar to Cho et al. [23]. We found the radiomic model outperformed the clinical model, which is similar to a recent study by Amudala Puchakayala et al. [17].

In our model, age, sex, height, and smoking status were selected as independent risk factors for identifying COPD. Smoking is one of the most common clinical risk factors, but a significant proportion of patients with COPD have never smoked [24]. Therefore, the model used smoking status as a surrogate for total smoking exposure in our study. Additionally, as in previous studies, age, sex, and height were independent predictors of COPD [2, 25, 26].

The AUC of the combined model with clinical and radiomic features was 0.893, 0.873, and 0.853 respectively, which was superior to the clinical model in the three cohorts, and slightly better than the radiomic model in the training and external validation cohorts. These findings are similar to the recent study published in *Radiology* [17], which indicated radiomic alone is a potent tool for identifying COPD. However, to provide a predicting tool for the probability of COPD occurrence at the individual level, we still constructed a nomogram based on the combined model. Compared with traditional methods, the nomogram can predict more quickly, conveniently, and accurately, especially it can help the physician to consult patients for health education.

Additionally, the diagnostic accuracy of the different models was assessed using an external validation cohort. The combined model showed slightly better than the radiomic model in the external validation cohort (*P* = 0.04), demonstrating that the model has good accuracy for this population. The results indicated the model has good predictive performance for new, unfitted data, as well as its high prediction capability and robustness. These findings are consistent with others utilizing radiomic to predict COPD survival [23, 27], spirometry-based evaluation of emphysema and severity [28], COPD exacerbations [29], COPD stage classification [30, 31], and analysis of COPD and resting heart rate [32].

Some limitations should be noted in this study. First, selection bias was inevitable due to its retrospective nature. The number of patients in the 5 centers was imbalanced, but the performance of the nomogram was good, confirming the universal applicability of our model. Second, only the CT radiomic features were evaluated, not common CT quantitative and qualitative parameters that are valuable in evaluating COPD. In future studies, we will extract common quantitative and qualitative parameters from paired inspiratory and expiratory CT images into our prediction model. Third, regarding the clinical variables, we only considered those that are most common and easy to acquire from CT scans. To more objectively evaluate the performance of the clinical model, future studies should incorporate more clinical variables, including symptoms. Fourth, the non-COPD patients in this study were defined as FEV1/FVC ≥ 0.7 and an FEV1% predicted ≥ 80% after bronchodilation, the preserved ratios of impaired spirometry (PRISm, FEV1/FVC ≥ 0.7 and an FEV1% predicted < 80%) were not included. PRISm is considered to be a high-risk factor for COPD, so it is very important to distinguish it from COPD. Our team will perform a tri-classification study.

## Conclusions

In conclusion, whole-lung CT radiomic can be used as a good biomarker for the identification of COPD, not only for the lung cancer screening population but also for all the patients who performed chest CT examinations. With the gradual development of AI technology, the quantitative and intuitive nomogram based on whole-lung CT radiomic may have wide clinical application value and more research should be performed toward automatic detection of COPD.

### Supplementary Information

**Additional file 1: Table S1 **CT protocols of the five centers. **Fig. S1** Typical lung region segmentation results from the original chest HRCT images segmented fully automatically and manually in the transverse, coronal, and sagittal planes. **Fig. S2** Boxplots show the whole lung CT radiomic signatures in COPD group were much higher than the non-COPD group in both the training (left) and test cohort (right). The calculation formula for the Radscore. The calculation formula for the combined model.

## Data Availability

All data generated or analyzed during this study are included in this article and its additional files.

## Abbreviations

AI: Artificial intelligence
AUC: Area under the curve
COPD: Chronic obstructive pulmonary disease
CT: Computed tomography
DCA: Decision curve analysis
FEV1/FVC: Ratio of forced expiratory volume in 1 s to forced vital capacity
FEV1% predicted: Percentage of predicted FEV1
LASSO: Least absolute shrinkage and selection operator
PFT: Pulmonary function test
ROI: Region of interest
