# Supplementary material for: CT whole lung radiomic nomogram: a potential biomarker for lung function evaluation and identification of COPD
Source: Mil Med Res. 2024 Feb 20;11:14. doi: 10.1186/s40779-024-00516-9 (PMC10877876; doi:10.1186/s40779-024-00516-9)
Supplement: Supplementary file 1 — Additional file 1: Table S1 CT protocols of the five centers. Fig. S1 Typical lung region segmentation results from the original chest HRCT images segmented fully automatically and manually in the transverse, coronal, and sagittal planes. Fig. S2 Boxplots show the whole lung CT radiomic signatures in COPD group were much higher than the non-COPD group in both the training (left) and test cohort (right). The calculation formula for the Radscore. The calculation formula for the combined model. [file 40779_2024_516_MOESM1_ESM.pdf]

Table S1 CT protocols of the five centers

Scanning equipment	Light speed 64VCT			Philips brilliance iCT				Philips ingenuity CT		Siemens SOMATOM			GE Optima CT	
	**Center 1**	**Center 4**	**Center 5**	**Center 1**	**Center 2**	**Center 3**	**Center 5**	**Center 1**	**Center 5**	**Center 2**	**Center 3**	**Center 4**	**Center 2**	**Center 4**
Tube voltage (kV)	120	120	120	120	120	120	120	120	120	120	120	120	120	120
Tube current (mAs)	30	30	30	AEC	AEC	AEC	AEC	AEC	AEC	200	200	200	200	200
Pitch	0.8	0.8	0.8	0.8	0.8	0.8	0.8	1.0	1.0	0.8	0.8	0.8	1.4	1.4
Collimation (mm)	0.6 × 64	0.6 × 64	0.6 × 64	0.6 × 128	0.6 × 128	0.6 × 128	0.6 × 128	0.6 × 128	0.6 × 128	0.8 × 64	0.8 × 64	0.8 × 64	0.6 × 64	0.6 × 64
Slice thickness (mm)	1	1	1	0.6/1	0.6/1	0.6/1	0.6/1	0.6/1	0.6/1	1/1.5	1/1.5	1/1.5	1.3	1.3
Matrix	512 × 512	512 × 512	512 × 512	512 × 512	512 × 512	512 × 512	512 × 512	512 × 512	512 × 512	512 × 512	512 × 512	512 × 512	512 × 512	512 × 512

Center 1: Tongji Hospital, School of Medicine, Tongji University, Center 2: Zhejiang Province People's Hospital, Center 3: Sir Run Run Shaw Hospital, Center 4: the First Affiliated Hospital of Nanchang Medical College, Center 5: the Second Affiliated Hospital of Naval Medical University. *AEC* dose modulation with automatic exposure control, *CT* computed tomography, *GE* general electric

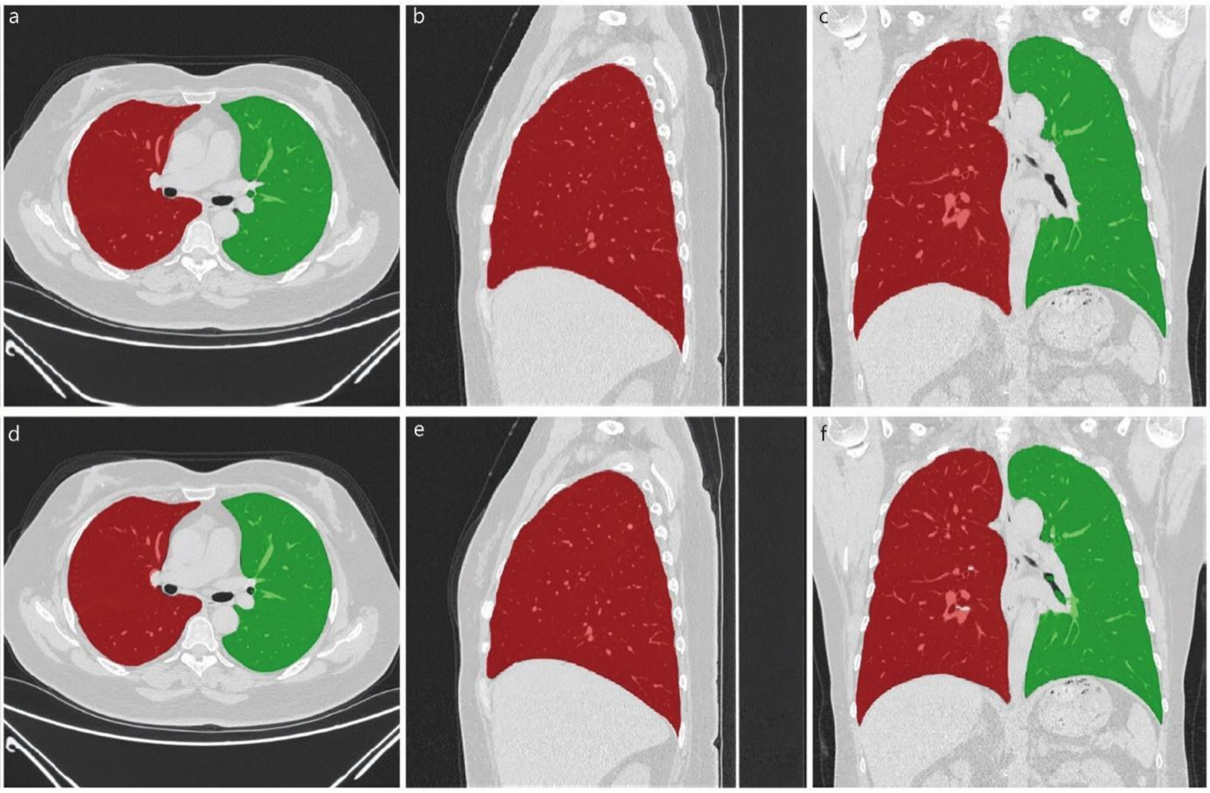

Fig. S1 Manual (**a-c**) and automatic (**d-f**) segmentation of typical lung regions in transverse, coronal, and sagittal planes based on the original chest HRCT images, respectively. The red mask is the right lung parenchyma, and the green one is the left. HRCT high-resolution computed tomography

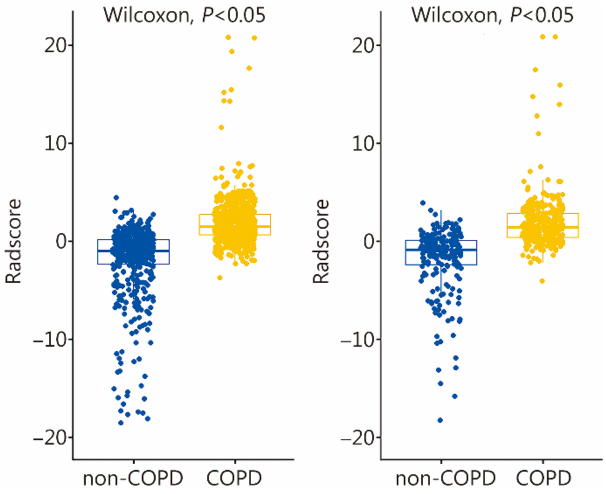

Fig. S2 Boxplots show the whole lung CT Radscores in COPD group was much higher than the non-COPD group in both the training (left) and test cohort (right). COPD chronic obstructive pulmonary disease, CT computed tomography

The calculation formula for the Radscore:

Radscore = 2.482 × wavelet_LLL_gldm_SmallDependenceLowGrayLevelEmphasis + (-0.564) × log_sigma_1.0_mm_3D_firstorder_RootMeanSquared + (-0.153) × wavelet_LLL_glcm_Correlation + 0.354 × wavelet_HHL_glszm_LargeAreaHighGrayLevelEmphasis + 6.117 × wavelet_LHL_glcm_ClusterShade + 0.026 × wavelet_HLH_firstorder_Mean + 0.704 × original_gldm_LowGrayLevelEmphasis + 0.646 × log_sigma_5.0_mm_3D_glcm_ClusterProminence + (-0.094) × wavelet_HHL_glrlm_RunVariance + (-1.248) × wavelet_LHL_firstorder_Mean + 0.178 × wavelet_LHL_glszm_LargeAreaHighGrayLevelEmphasis + 0.756 × wavelet_LLL_glcm_Imc2 + (-0.433) × original_shape_Flatness + 0.341 × log_sigma_5.0_mm_3D_firstorder_Maximum + 0.096 × log_sigma_5.0_mm_3D_glcm_Imc2 + (-0.083) × wavelet_HLL_firstorder_Skewness + (-2.19) × wavelet_LLL_gldm_LowGrayLevelEmphasis + (-0.825) × log_sigma_1.0_mm_3D_firstorder_Median + 0.389

The calculation formula for the combined model:

Nomoscore = (Intercept) × (-4.744) + Age × 0.024 + Height × 0.021 + Smoking × (-0.412) + Gender × 0.609 + Radscore × 1.026
